# Resveratrol supplementation rescues pool of growing follicles and ovarian stroma from Cisplatin-induced toxicity on the ovary in Sprague-Dawley rats: An experimental study

**Published:** 2018-01

**Authors:** Gbotolorun Stella Chinwe, Okafor Izuchukwu Azuka, Ndoeche Chidinma Adaeze

**Affiliations:** *Anatomy Department, College of Medicine of the University of Lagos, Lagos State, Nigeria.*

**Keywords:** Cisplatin, Ovary, Resveratrol, Sprague-Dawley rat

## Abstract

**Background:**

Cisplatin is a potent antineoplastic agent for many cancers but causes several levels of gonadal damage. Ovarian toxicity is a major concern of young cancer patients undergoing chemotherapy.

**Objective:**

This study sought to examine the effect of Cisplatin and Resveratrol supplementation on ovarian function in Sprague-Dawley rats.

**Materials and Methods:**

In this experimental study, 45 cyclic Sprague-Dawley rats with an average weight of 160 gr were divided into 9 groups (n=5/group). Group 1 was used as control and received distilled water. Groups 2 and 9 received Cisplatin only. Groups 3, 4, and 5 received different doses of Resveratrol after a single dose of Cisplatin. Groups 6, 7, and 8 received Resveratrol before Cisplatin. At sacrifice, the ovary was analyzed for histopathology, biochemical indices of oxidation and hormonal assay.

**Results:**

Relative and absolute organ weights were notably increased (p=0.001, 0.01) in the prophylactic groups relative to the groups that received Resveratrol after Cisplatin. Also, glutathione, superoxide dismutase and catalase were significantly increased (p=0.047, 0.01, 0.023) in a dose-dependent manner when compared to Cisplatin group only. Malondialdehyde decreased significantly (p=0.001) in the groups that received high dose Resveratrol compared with the control and Cisplatin alone groups. Although oestrogen showed no significant difference within the groups (p=0.48), Resveratrol significantly increased progesterone, follicle stimulating hormone and luteinizing hormone levels (p=0.007, 0.001, 0.006) at high doses when compared with Cisplatin alone groups. Ovarian histoarchitecture was best preserved in the prophylactic groups in a dose-dependent manner.

**Conclusion:**

Resveratrol supplementation confers protection and preserves ovarian follicles from Cisplatin toxicity in Sprague-Dawley rats.

## Introduction

Resveratrol (trans-3, 5, 4-trihydroxystilbene) is a phytoalexin, which belongs to the group of antibiotic compounds (1). Injury, stress, bacteria or fungi infection, UV-irradiation and exposure to ozone foster their production as part of the plant’s defense system (1, 2). A recent review noted that Resveratrol is present in almost negligible amount in human diet, and if Resveratrol or similar compounds are proven useful, a supplement or drug rather than diet is likely to be the source (3).

Cisplatin has been used for various cancer treatments including that of the liver but particularly effective against testicular cancer. Although as chemotherapeutic agents for cancer treatment, Cisplatin has produced positive outcomes however; the toxic effects that it generates often interferes with its therapeutic efficacy (4). The exact mechanism by which Cisplastin produces these side effects are still not clearly understood but researchers strongly opine that it is not by one factor but rather by a combination of multiple factors (5). To prevent these undesirable side effects, most studies have relied largely on the use of possible preventive agents as supplements with Cisplatin treatment (6).

Resveratrol has been reported to possess anti-inflammatory, antioxidant, analgesic, neuroprotective, antiviral and anti-aging actions. Its cardio-protective effect and platelet aggregation inhibition have been well documented (7). Resveratrol can exhibit both antioxidant and pro-oxidant abilities and this is dependent largely on the dose administered hence its documented roles in reducing oxidative stress (2).

Animal studies using Resveratrol have reported its antitumor, antidepressant and antidiabetic effects (8-10). In vivo study showed that Resveratrol can improve sperm count and sperm quality, improve testosterone levels and also prevent sperm DNA damage resulting from cryopreservation in humans (11). 

There may be a hidden potential in Resveratrol to act as a possible supplementation attenuating the Cisplatin-induced deleterious effect on the female reproductive organs and function. Hence, the need to elucidate the beneficial or otherwise effect of Cisplatin and Resveratrol supplementation on the reproductive function of the female Sprague-Dawley rats.

## Materials and methods

**Care and handling of Animal**

This experimental study was carried out in the research laboratory of the Department of Anatomy, University of Lagos, Nigeria from July 2016 to February 2017. The rats were procured from the Animal House, College of Medicine of the University of Lagos. They were acclimatized for 2 wk to exclude any intercurrent infection under standard housing of 24±2^o^C and 12 hr light/dark cycles. The animals were given standard rat chow and water ad-libitum. 

**Experimental drugs**

Resveratrol with the brand name ‘Restorlyf’, manufactured by Nature’s Way U.S.A., was procured from Alliance in Motion Global Ltd., Ikeja, Lagos, Nigeria. 325 mg of Resveratrol were diluted immediately before each use in 20 ml of distilled water and the doses of 5, 10, and 20 mg/kg/b.wt. were administered orally using the oral cannula. The remaining formulation was discarded after each use. The drug dosages and formulations were chosen on the basis of previously published studies on Resveratrol (13, 14).Cisplatin (Zuplatin, 50 mg/ 50 ml) injection manufactured by Taj pharmaceuticals Ltd. India was procured from Bayston Pharmacy, Mushin, Lagos, Nigeria. The injection was given intraperitoneally and according to the animal weight in a single dose of 5 mg/kg. The drug dosage was chosen on the basis of previously published studies on Cisplatin (15, 16).

**Experimental design**

Forty-five adult cyclic Sprague-Dawley rats averagely weighing 160 gr divided into 9 groups (n=5/group) were used. Group 1 was control group and was fed distilled water only. Group 2 was given only a single dose intraperitoneal injection of 5 mg/kg b.wt. Cisplatin and allowed to stay for 7 days before sacrifice. Groups 3, 4, and 5 were given 5, 10, and 20 mg/kg/b.wt. Resveratrol respectively for 7 days, starting 24 hr after a single dose intraperitoneal injection of 5 mg/kg/b.wt. Cisplatin. Groups 6, 7, and 8 were treated with 5, 10, and 20 mg/kg/b.wt. Resveratrol respectively for 14 days before a single dose intraperitoneal injection of 5 mg/kg b.wt. Cisplatin; the respective doses of Resveratrol treatment was repeated for another 7 days. Group 9 was given only a single dose intraperitoneal injection of 5 mg/kg b.wt. Cisplatin and allowed to stay for 21 days before sacrifice. At the end of the study, body weight, absolute, and relative ovary weights were accessed; the ovary was accessed for histopathology and oxidative stress including Glutathione (GSH), Superoxide dismutase (SOD), Catalase (CAT) and Malondialdehyde (MDA). 

**Animal sacrifice and sample collection**

On conclusion of the study, the animals were not fed on the night before they were to be sacrificed the next day, and sacrifice was by cervical dislocation. Blood samples were collected by cardiac puncture into plain bottles for hormonal assay. The serum was separated by allowing the blood sample to stand for 15 min at 25^o^C and then centrifuged at 4000 rpm for 20 min. Serum was kept in plastic vials at -40^o^C until further biochemical analysis. The ovaries were excised, trimmed of fat and weighed immediately before rinsing in cold saline and the right ovary of each rat was immediately stored at -20^o^C for oxidative stress assay while the left ovary was fixed in 10% formal saline for histological preparation.

**Tissue processing**

Seventy-two hr after fixation, the tissues were dehydrated by passing through ascending grades of alcohol. Tissues were cleared in xylene after dehydration and then embedded in molten paraffin wax at a temperature of 60^o^C. The issues were then sectioned at 5 µm and stained with haematoxylin and eosin. Photomicrographs of these sections were obtained using the DM 750 Leica digital photomicroscope. Atretic follicles, antral follicles and primordial follicles were evaluated in the tissue micrographs. The techniques described by Sadrkhanloo and his colleagues were used (17).

**Biochemical analysis of oxidation**

The ovarian tissue was homogenized in a Teflon-glass homogenizer with a buffer containing 1.5% potassium chloride to get the 1:10 (w/v) whole homogenate. MDA was measured using the thiobarbituric acid test to determine the concentrations of ovarian MDA level. The reduced GSH and Catalase levels in ovarian tissue were estimated as described by Rukkumani and his colleagues while SOD activity was measured by the method described by Sun and colleagues (18, 19).

**Hormonal assays**

AccuBind enzyme-linked immunoabsorbent assay (ELISA) microwells for Estradiol, Progesterone, follicle stimulating hormone (FSH) and luteinizing hormone (LH) were purchased from Monobind Inc. Lake Forest CA USA with the respective product codes-4925-300, 4825-300, 425-300, and 625-300. To minimize the probable effect of diurnal influences on hormones, samples were collected in the morning. The reagents, serum references and control were brought to room temperature before the assay. AccuBind Procedure was used for the assessment of FSH, LH, Estradiol, and progesterone (20). The laboratory technician was unaware of the treatment allocation.

**Ethical consideration**

Ethical approval was gotten from the College of Medicine of the University of Lagos Health Research Ethics Committee (CMULHREC) with ID number CMULHREC/ 09/16/025. All procedures were carried out in accordance with the National Academy of Science’s Guide for Care and Use of Laboratory Animals (12).

**Statistical analysis**

The results were analyzed using the Statistical Package for Social Sciences, version 17.0, SPSS Inc., Chicago, Illinois, USA (Statistical Package for the Social Science). Data was reported as mean±SEM differences between mean and the main effects of the treatment group were determined by the one-way analysis of variance (ANOVA) and multiple comparisons were done using the LSD post-hoc tests. The mean difference is significant at the 0.05 level (p<0.05).

## Results

**Body weight measurement**

There is a notable difference (p=0.01) between the pre and post-administration body weight in Groups 1, 4, and 5. All other groups (2, 3, and 6-9) showed no difference in the body weight before and after administration (p=0.48). Group 4 experienced the highest decrease in percentage body weight within all the groups (Table I).

**Ovarian weight**

Groups 2-5 revealed a notable decrease (p=0.01) when absolute weight of the ovaries were compared with control group while all other groups were shown not to be significantly different from control group. There was a significant decrease in the absolute ovarian weight of groups 1, 6, 7, 8, and 9 when compared with group 2. Groups 2-5 significantly decreased (p=0.00) in relative ovary weight in comparison with the control group. Groups 1 and 6-9 were significantly higher (p=0.00) than group 2 while groups 2, 3, 4, 5, and 8 significantly reduced (p=0.05) in comparison to group 9 (Table I).

**Oxidative status**

All treatment groups showed no significant difference in GSH Levels when compared to groups 1, 2 and 9 except for group 8 which showed a significantly higher (p=0.05) GSH level than groups 2 and 9. No significant difference in SOD was observed when treated groups were compared to control. Groups 8 was noted to be significantly higher (p=0.01) than the Cisplatin control groups. All treatment groups showed no significant difference in ovarian Catalase compared to groups 1, 2 and 9 (p=0.67). MDA was significantly higher (p=0.001) in group 9 compared to the control group. Groups 3, 4, and 5 had a significant decrease (p=0.001) in MDA when compared with groups 2 and 9. Group 6 showed a significant decrease (p=0.001) when compared with group 9 (Table II).

**Hormone assay**

There was a notable decrease in progesterone levels of groups 2 and 7 when compared with the control group (p=0.007, 0.01). No significant change was observed when the other groups were compared with control (p<0.05). Estradiol levels did not experience any significant change when all the other treatment groups were compared with the control group and Cisplatin groups (Table III).

LH increased significantly (p=0.006) in groups 3 and 5 when compared with both the control group and group 2; only group 5 and 6 showed an increase in LH level when compared to group 2 (p=0.006) while group 6 showed an increased level of LH when compared with group 2 only (Table III).

Groups 2, 3, 4, 6, 8, and 9 showed a significant decrease (p=0.00) in FSH levels compared to the control. The control group and groups 4, 5, 6, 7 and 8 showed a significant increase (p=0.05) in FSH levels when compared with groups 2 and 9 (Table III).

**Histological Observation**

Histological sections from the control group demonstrated normal ovarian histoarchitecture with ovarian follicles at different stages of development. The rats treated with Cisplatin only, showed the presence of atretic follicles with basement membrane distortions and separation of theca folliculi from the granulosa cells. Cisplatin toxicity equally manifested as thinning of the cumulus oophorus, oocyte degeneration, stromal fibrosis and fibrolysis of the corpus luteum. Supplementation with low dose Resveratrol (5 mg/kg/b.wt.) given after single dose Cisplatin (5 mg/kg) treatment offered a mild protection as there were still a minor loss of cumulus cells and cortical distortions. 

In addition, supplementation with low dose Resveratrol (5 mg/kg/b.wt.) before and after a single dose Cisplatin injection for 21 days equally did not offer full protection against Cisplatin ovarian toxicity. The ovarian tissue sections showed minor displacement of granulosa cells and attrition of the theca externa. Photomicrograph of rats that received medium dose (10 mg/kg/b.wt.) supplementation of Resveratrol after single dose Cisplatin (5 mg/kg/b.wt) injection showed a near normal ovarian histoarchitecture with the presence of preovulatory follicle and full preservation of theca folliculi (**Figure 8**). These same effects were seen in the photomicrographs of rat ovary supplemented with medium-dose Resveratrol both before and after Cisplatin (5 mg/kg/b.wt.) injection for 21 days. 

High dose (20 mg/kg/b.wt.) Resveratrol supplementation for 7 days given after single dose (5 mg/kg/b.wt.) Cisplatin injection showed a normal ovary histoarchitecture with the presence of good pool of varied stages of growing follicle. A 21-day supplementation of high dose Resveratrol before and after the single dose (5 mg/kg/b.wt.) Cisplatin equally showed a completely normal ovarian histoarchitecture. Resveratrol at a high dose showed maximum protection on the rat ovary against Cisplatin toxicity hence increasing its fertility potential. 

**Table I:** The effect of Cisplatin and supplementation with Resveratrol on the body weight of female Sprague-Dawley rats

**Groups**	**Pre-administration body weight (g)**	**Post-administration body weight (g)**	**Body weight** **difference (%)** *	**Relative ovarian weights (×10-4 )**	**Absolute ovarian weights (g)**
Group 1	136.6 ± 1.6	151.0 ± 4.0 ^β^	9.5	6.6 ± 0.03^b^, ^c^	0.10 ± 0.03^b^
Group 2	217.2 ± 1.6	214.8 ± 1.2	1.1	2.5 ± 0.02^a^, ^c^	0.05 ± 0.01^a^, ^c^
Group 3	171.0 ± 11.5	156.2 ± 14.3	9.5	4.2 ± 0.01^a^,^b^, ^c^	0.07 ± 0.01^a^, ^c^
Group 4	148.4 ± 3.2	123.8 ± 6.8 ^β^	19.9	4.4 ± 0.01^a^,^b^,^ c^	0.05 ± 0.00^a^, ^c^
Group 5	148.6 ± 2.8	125.4 ± 1.0 ^β^	18.5	4.3 ± 0.02^a^,^b^,^c^	0.05 ± 0.02^a^,^c^
Group 6	160.2 ± 2.5	167.0 ± 7.4	4.1	6.3 ± 0.03^b^,^c^	0.11 ± 0.01^b^
Group 7	147.4 ± 0.7	142.0 ± 4.9	3.8	6.8 ± 0.02^b^,^c^	0.10 ± 0.01^b^
Group 8	149.6 ± 2.1	143.0 ± 3.9	4.6	6.0 ± 0.01^b^,^c^	0.09 ± 0.01^b^,^c^
Group 9	169.4 ± 2.4	156.2 ± 5.9	8.5	7.7 ± 0.01 b	0.12 ± 0.00^b^

Values are expressed as mean ± S.E.M. β p<0.05 significant difference between pre and post administration body weights.

a : p<0.05 significant compared to control group

b : p<0.05 significant compared to group 2

c : p<0.05 significant compared to group 9

* The mean difference between pre and post administration body weights in each group

**Table II:** The effect of Cisplatin and supplementation with Resveratrol on the oxidative stress markers of the ovarian tissue in Sprague-Dawley rats

**Group**	**GSH (µmol/ml)**	**SOD (µmol/ml/min/mgpro)**	**CATALASE (µmol/ml/min/mgpro)**	**MDA (µmol/ml)**
Group 1	2.1 ± 0.1^b^,^c^	5.2 ± 0.2^b^, ^c^	48.5 ± 6.2 b, c	0.3 ± 0.1 b, c
Group 2	0.5 ± 0.2^a^	4.1 ± 0.1 a	39.1 ± 8.2 a	0.5 ± 0.1 a
Group 3	2.3 ± 0.2	5.1 ± 0.9	44.5 ± 4.5	0.5 ± 0.1 a
Group 4	0.7 ± 0.2	4.5 ± 0.9	52.8 ± 7.5 b, c	0.2 ± 0.1 b, c
Group 5	0.5 ± 0.2	4.6 ± 1.2	60.8 ± 8.9^a^, ^b^, ^c^	0.1 ± 0.0 a, b, c
Group 6	0.4 ± 0.2	5.1 ± 0.7	45.2 ± 3.2	0.3 ± 0.1 c
Group 7	1.2 ± 0.0^b^,^c^	6.8 ± 1.9^b^, ^c^	59.3 ± 3.3 a, b, c	0.5 ± 0.2
Group 8	4.7 ± 0.3^a^,^b^,^c^	8.3 ± 1.6^a^,^b^, ^c^	60.1 ± 3.2 a, b, c	0.1 ± 0.0^a^, ^b^, ^c^
Group 9	0.5 ± 0.1^a^	4.2 ± 0.4 a	36.2 ± 4.5^a^	0.6 ± 0.0 a

Values are expressed as mean±SEM

GSH: Glutathione.

SOD: Superoxide dismutase

MDA: Malondialdehyde.

a : p<0.05 significant compared to control group

b : p<0.05 significant compared to group 2

c : p<0.05 significant compared to group 9

**Table III:** The effect of Cisplatin and supplementation with Resveratrol on the reproductive hormones of female Sprague-Dawley rats

**Groups**	**Progesterone (ng/ml)**	**Estradiol (pg/ml)**	**LH (miu/ml)**	**FSH (miu/ml)**
Group 1	27.8 ± 6.3^b^	57.0 ± 10.1	1.1 ± 0.2	3.26 ± 0.7 b, c
Group 2	9.8 ± 2.4^a^	36.0 ± 6.7	0.4 ± 0.2	0.26 ± 0.0 a
Group 3	23.8 ± 4.7^b^	35.7 ± 5.3	4.7 ± 2.3 a, b	0.96 ± 0.2 a, b
Group 4	22.0 ± 3.8	39.1 ± 5.1	2.0 ± 0.7	1.09 ± 0.2 a, b, c
Group 5	16.3 ± 3.3	37.9 ± 1.6	6.4 ± 1.9 a, b, c	3.01 ± 0.3 b, c
Group 6	15.0 ± 5.3	40.2 ± 8.4	4.8 ± 1.4 b	2.33 ± 0.0 a, b, c
Group 7	10.7 ± 0.9^a^	54.0 ± 15.0	2.3 ± 1.4	2.63 ± 0.1 b, c
Group 8	23.5 ± 7.9^b^	61.2 ± 12.6	0.9 ± 0.2	2.47 ± 0.2 a, b, c
Group 9	22.4 ± 3.6	37.4 ± 4.6	1.5 ± 0.9	0.4 ± 0.0^a^

Values are expressed as mean±SEM

LH: Luteinizing hormone

FSH: Follicle stimulating hormone

a : p<0.05 significant compared to control group

b : p<0.05 significant compared to group 2

c : p<0.05 significant compared to group 9

**Figure 1:**
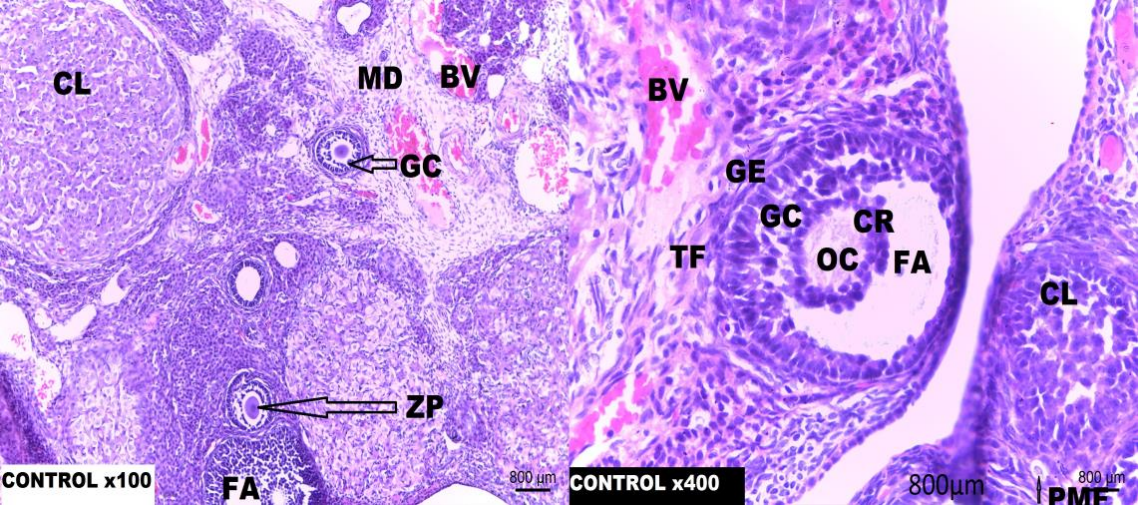
Photomicrograph of rat ovary tissue section of Group 1.

H & E section of rat uterus (×10) treated with distilled water for 21 days showing normal ovarian histoarchitecture. Normal growing follicles are seen: Primary follicle (PF), secondary follicle and antral follicle with a follicular antrum (FA). Zona pellucida (ZP) can be seen surrounding the oocyte (OC). Growing follicles shows granulosa cells (GC) surrounding them. Medulla (MD) and corpus luteum (CL) can also be seen. In the H & E section of rat ovary (×400), normal preovulatory follicles are seen with large follicular antrum (FA) and the cumulus oophorus (CO) and corona radiate (CR) surrounding the oocyte (OC). A normal cortical stroma can be seen with the theca folliculi (TF) surrounding the granulosa cells (GC).

**Figure 2:**
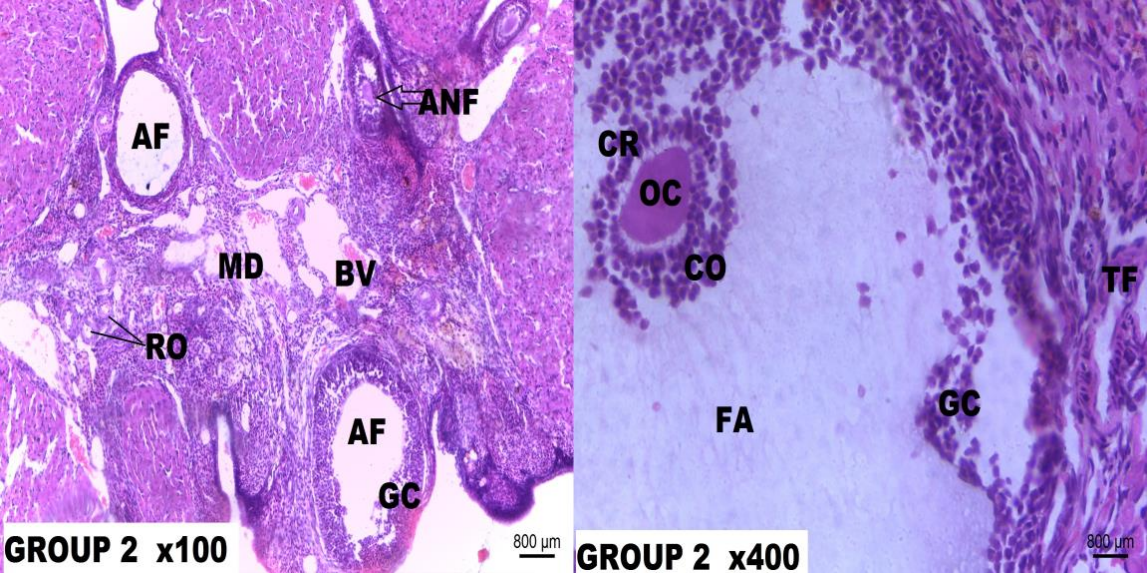
Photomicrograph of the rat ovary tissue of Group 2.

**Figure 3.**
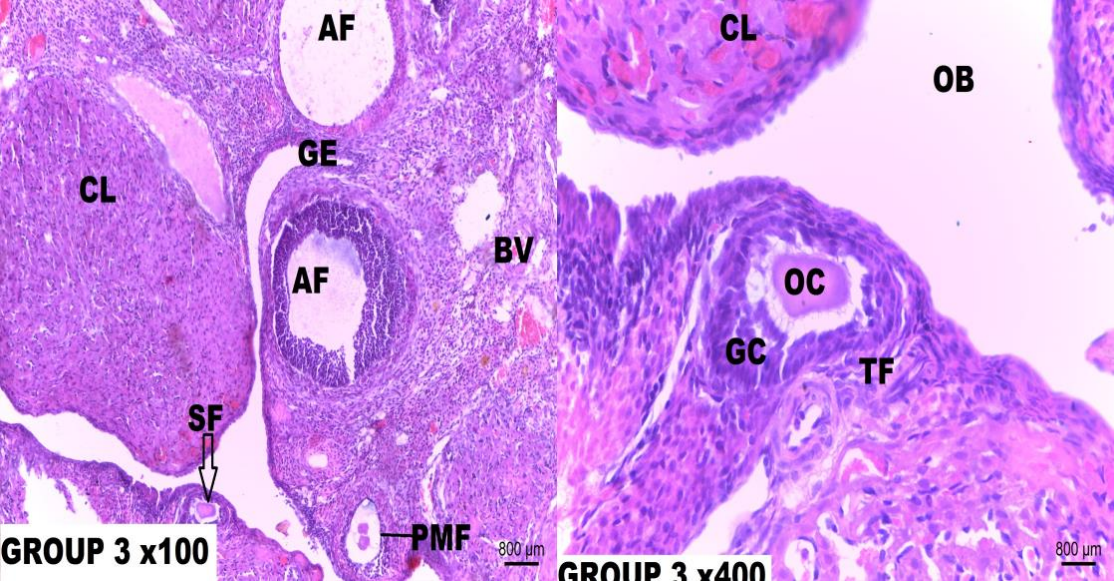
Photomicrograph of rat ovary tissue section of group 3

**Figure 4:**
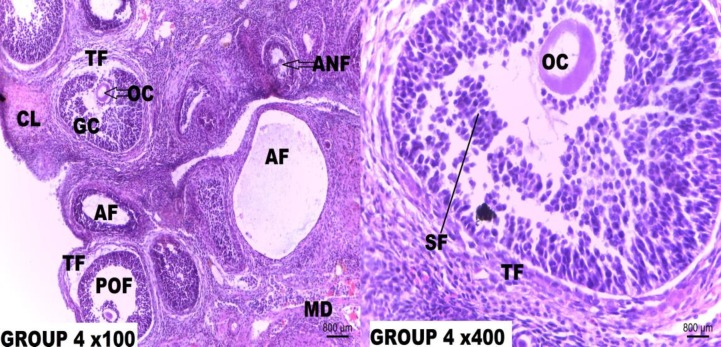
Photomicrograph of the rat ovary tissue section of Group 4.

**Figure 5:**
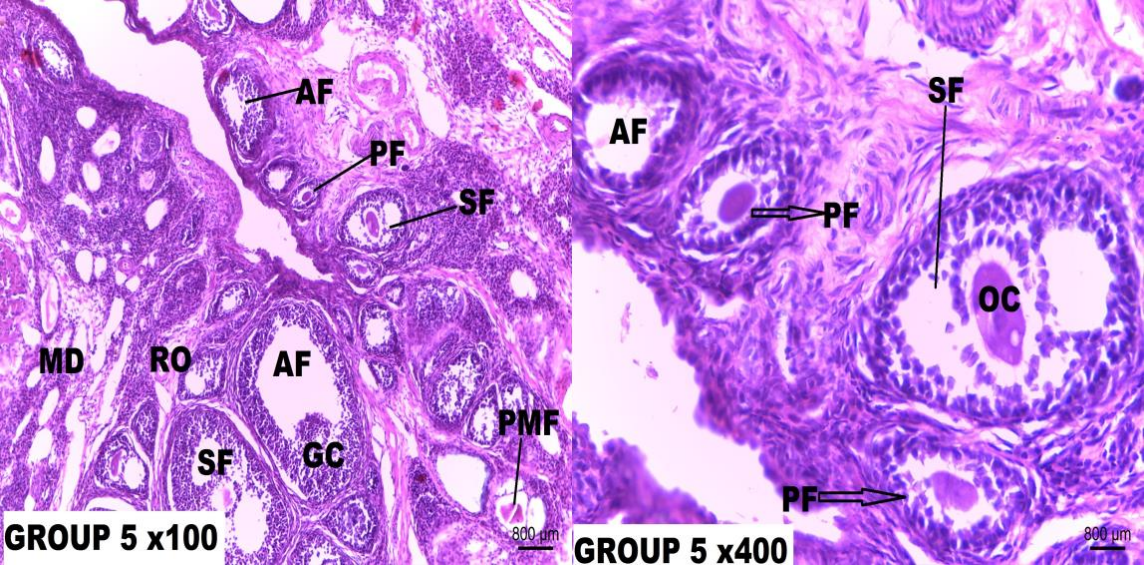
Photomicrograph of rat ovary tissue section of Group 5.

## Discussion

Resveratrol, being a natural phenol, has been investigated and observed to be effective in several forms of diseases including cancer and female infertility (21-23). This has been attributed to its role as an antioxidant and anti-apoptotic agent (22, 23). The result of this study showed that Cisplatin significantly reduced body weight of the animals (p=0.01) but this body weight loss has been prevented across all groups treated with Resveratrol except for groups treated with medium and high dose Resveratrol after Cisplatin injection. This shows Resveratrol to be effective in weight loss prevention after Cisplatin chemotherapy. The effect may be due to its reported role in glucose metabolism in disease states (24). In another study, Resveratrol, over prolonged duration was observed to cause fat loss in obese patients. Body weight measurements observed in this study was similar to the findings in the study reported by Helal and his colleagues (25).

Organ-body weight measurements have been used to determine toxicity level in tissues (26). The relative ovarian weight of the prophylactic groups (groups 6, 7 and 8) was not different significantly (p<0.05) from the control group but were significantly higher than the Cisplatin control group: group 2 (Table I). This study showed a prophylactic function of Resveratrol against the Cisplatin-induced decrease in relative ovarian weight (p=0.00). However, Resveratrol failed to maintain or increase the relative ovarian weight of post-treatment groups (groups 3, 4 and 5) in comparison with control (p<0.05). The prophylactic Resveratrol treatment groups (6-8) were also seen to have significantly higher absolute ovarian weight than the group 2 (p=0.01).

While examining the oxidative stress markers in a chemotherapy-induced gonadotoxicity model, Helal and colleagues (25) found significantly increased MDA with significantly decreased SOD and CAT in ovarian tissue of anticancer treated groups. However, Pinar and colleagues observed no significant difference in ovarian levels of MDA and SOD in animal groups treated with Cisplatin alone and Resveratrol before Cisplatin injection (23).

In this study, reduced GSH, CAT, MDA and SOD were observed (Table II). GSH levels in the ovaries showed no appreciable change when the treated groups were compared to control however, in the prophylactic group that received high dose Resveratrol and the Cisplatin only group (group 2), GSH recorded significant increases compared to control. It is interesting to note that same effect was seen in the ovarian SOD levels. Group 7 equally showed significant increases in ovarian SOD levels in comparison with groups 2 and 9; its increase is not significant when compared to control. CAT levels of the ovarian tissues recorded significantly high activities both of the medium and high dose Resveratrol post-treatment groups (group 4 and 5) and medium and high dose Resveratrol prophylactic groups (groups 7 and 8) when compared with Cisplatin-treated groups (group 2 and 9) (p<0.05); only groups 5, 7 and 8 showed significant elevations in ovarian CAT levels in comparison with the normal control group (p=0.02). Howbeit, it has been demonstrated that some pathologies causing sub-fertility or infertility in both males and females result from oxidative stress because of the deleterious effect of oxidative stress generated in human reproduction (27). Resveratrol has a documented role in reducing oxidative stress acting as an antioxidant or pro-oxidant (2). 

In this study, prophylactic intervention with the high dose of Resveratrol has shown to have played a role in increasing both GSH, SOD and CAT significantly (p=0.047, 0.01, 0.023). However, the potential protective effect of antioxidants against tissue damage related to oxidative stress is unclear (28). Increase in antioxidant status by Resveratrol has been observed by many researchers in different study models with SOD and MDA mostly favored (29-31).

Lipid peroxidation was investigated in this study using the MDA (Table II). Resveratrol-treated groups were able to significantly reduce MDA levels in the Resveratrol high dose post-treated and high dose prophylactic groups (p=0.00) compared to normal control groups and the Cisplatin only groups. Lipid peroxidation is the most notable sign of cell damage, causing change of membrane polyunsaturated fatty acids to hydroperoxides and further degradation to low molecular species. However, this study is in consonance with the findings of Bradamante and colleagues who reported Resveratrol to have antioxidant properties that is able to reinforce endogenous cellular antioxidant systems and mop up ROS (32).

In this study, progesterone (PG), Estradiol (EST), FSH and LH levels were assayed (Table III). There was no significant difference in EST level of Resveratrol-treated groups when compared with the normal control group and Cisplatin control groups (p=0.48). However, disruption of ovarian function by anticancer drugs may present with low EST levels though this effect may be partial (27). EST expression has been observed in some specific tissues including the ovary, uterus and breast (33) and it is the prevalent oestrogen during reproductive years both in terms of absolute serum levels as well as in terms of oestrogenic activity (34). The non-significant decrease in estrogen observed in the groups treated with Cisplatin only, may be as a result of estrogen modulation by inhibition of its receptors and expression as reported by Otto and colleagues (35). This study, however, reveals that Resveratrol has the ability to prevent undue estrogen modulation thereby maintaining estrogen at normal physiological levels.

Receptors of PG are also being inhibited by Cisplatin (35). Since eggs release PG in the ovary, the number of eggs present in the ovary may be correlated to the amount of PG concentration; more so, if corpus luteum does not develop, levels of PG may be low (36, 37). In this study, PG concentrations were found to be decreased significantly in the groups of animals treated with Cisplatin only when compared with the normal control (p=0.01) (Table III). Moreover, all the groups treated with Resveratrol appeared not to be significantly different to the normal control group except for the medium-dose prophylactic Resveratrol group (p<0.05). This may suggest Resveratrol’s ability to maintain the PG concentrations of female rats during Cisplatin chemotherapy at physiologically normal levels.

Ovary disorder such as ovarian failure is sometimes confirmed biochemically by elevated FSH. However, the drawback with this marker is that it can only detect the endpoint of ovarian function decline but cannot predict future reproductive potential and moreover, it is not a sensitive marker of ovarian reserve after exposure to chemotherapy (38, 39). Though high FSH levels are indications of sub-fertility and/or infertility, they are also important for the recruitment and further development of immature ovarian follicles in the ovary (40). 

In fact, low FSH levels have been implicated in polycystic ovarian syndrome and impaired gonadal function (40). Strumberg and his colleagues (41), reported an increase in FSH and LH after Cisplatin based chemotherapy treatment, while a study by Helal and colleagues (25) showed a significant decrease in FSH and LH levels in the combination chemotherapy model. The result of this study showed a significant decrease in FSH levels of Cisplatin-treated rats and rats treated with Cisplatin and Resveratrol when compared to the normal control group (p=0.001). Moreover, there were significantly higher FSH levels in Prophylactic groups of Resveratrol when compared with the Cisplatin only group (p=0.000) (Table III). The mechanism behind this hormone modulation by Resveratrol remains unknown but suggests an intrinsic modulating ability of Resveratrol on FSH levels. 

In humans, LH is said to work together with FSH and its levels remain almost same with FSH except towards the mid ovarian cycle when there is an ‘LH surge’ (40, 42). Investigation on the LH in this study revealed that LH levels, when compared to the control group, increased significantly in only low and high dose prophylactic Resveratrol-treated groups (p=0.01) but was not altered significantly in all other Resveratrol treated groups. When compared to the ‘Cisplatin only’ treatment groups, Resveratrol showed significant ability to increase LH levels in low and high dose Resveratrol treated groups (p=0.01) (Table III). It is not still clear on the mechanism of modulation of LH in Cisplatin treatment by Resveratrol as seen in this study.

In this study, the histological sections of the normal control group showed normal ovarian histoarchitecture with ovarian follicles at different stages of development. The rats treated with Cisplatin only showed the presence of atretic follicles with basement membrane distortions, separation of theca folliculi from the granulosa cells. Cisplatin toxicity equally manifested as thinning of the cumulus oophorus, oocyte degeneration, stromal fibrosis and fibrolysis of the corpus luteum. Research findings have attributed a wide variety of biological property to Resveratrol including anti-inflammatory, antioxidant, anti-proliferative, pro-apoptotic and anticancer effects (43, 44). Antioxidants are involved in reducing follicular atresia connected with apoptosis and reactive oxygen species damage (44).

The low-dose Resveratrol (5 mg/kg/b.wt.) supplementation given after single dose Cisplatin (5 mg/kg) treatment offered a mild protection as there are still a minor loss of cumulus cells and cortical distortions. More so, supplementation of low dose Resveratrol (5 mg/kg/b.wt.) before and after single dose Cisplatin injection for 21 days equally did not offer full protection against Cisplatin ovarian toxicity. The ovarian tissue micrograph presents minor displacement of granulosa cells and attrition of theca externa. Photomicrographs of rats that received a supplementation of medium-dose (10 mg/kg/b.wt.) Resveratrol after single dose Cisplatin (5 mg/kg/b.wt) injection showed a near normal ovarian histoarchitecture with presence of preovulatory follicle and full preservation of theca folliculi. These same effects were seen in the photomicrographs of rat ovary supplemented with med-dose Resveratrol both before and after Cisplatin (5 mg/kg/b.wt.) injection for 21 days. 

However, high dose (20 mg/kg/b.wt.) Resveratrol supplementation for 7 days given after single dose (5 mg/kg/b.wt.) Cisplatin injection showed a normal ovary histoarchitecture with the presence of a good pool of varied stages of growing follicle. A 21-day supplementation of high dose Resveratrol before and after the single dose (5 mg/kg/b.wt.) Cisplatin equally showed a completely normal ovarian histoarchitecture. Resveratrol at a high dose showed maximum protection on the rat ovary against Cisplatin toxicity hence increasing its fertility potential. This study agrees with the findings of Kong and his colleagues who reported that Resveratrol significantly increased the number of oocytes, decreased atretic follicles and inhibited follicular apoptosis in ovaries of different age groups (45). Pinar and his colleagues opined that these effects may be associated with increased telomere length and telomere activity and reduction of germ cell function (23).

Ortega and other colleagues showed that Resveratrol stimulates proliferation of rat granulosa cells and exhibiting pro-apoptotic actions on theca-interstitial cells hence they suggested Resveratrol as a novel treatment modality for polycystic ovarian syndrome; however, their findings agree with the findings of this study (46). 

Already characterized oxidative stress induced histopathological changes were seen in this study, hence, it can be inferred that the Resveratrol effect on ovarian Cisplatin toxicity may be by inhibition of the tissue oxidative stress. Moreover, this study may also suggest a possible correlation between the effects of Resveratrol on the ovary as demonstrated histologically, to its hormonal influence and antioxidative output. Howbeit, Resveratrol’s role in ovarian Cisplatin toxicity may be by a combination of multiways.

## Conclusion

Resveratrol maintains ovarian architecture and prevents oxidation by increasing ovarian SOD and GSH levels with modulations of FSH and LH concentrations. Also, this study provided substantial evidence of Resveratrol’s attenuation and prevention of Cisplatin-induced ovarian toxicity in Sprague-Dawley rats.
